# Isoperistaltic gastric tube for long gap esophageal atresia (LGEA) in newborn, infants, and toddlers: a case-control study from a tertiary center

**DOI:** 10.3389/fped.2023.1194928

**Published:** 2023-05-16

**Authors:** Angelo Zarfati, Renato Tambucci, Pietro Bagolan, Andrea Conforti

**Affiliations:** ^1^ Department of Systems Medicine, University of Rome “Tor Vergata”, Rome, Italy; ^2^ Neonatal Surgery Unit, Bambino Gesù Children’s Hospital, IRCCS, Rome, Italy; ^3^ Digestive Endoscopy and Surgery Unit, Bambino Gesù Children’s Hospital, IRCCS, Rome, Italy

**Keywords:** esophagal atresia, long-gap atresia, long-gap, gastric tube esophagoplasty, gastric tube, isoperistaltic gastric tube, anastomosis

## Abstract

**Background:**

Limited evidence exists about outcomes after gastric tube formation as “rescue” technique to avoid esophageal replacement in long gap esophageal atresia (LGEA). The last ERNICA Consensus Conference on the Management of LGEA has placed the techniques of gastric tubulization among the priorities for future research.

**Aims:**

Evaluate personal experience with Isoperistaltic Gastric Tube (IGT) and compare its outcomes with other more popular techniques for LGEA.

**Methods:**

A case-control study has been conducted. A retrospective monocentric analysis of LGEA patients (period: 2010–19) has been conducted in all consecutive IGT patients and each of these has been type matched with two cases of LGEA treated with other techniques. The follow-up (FU) considered was 24-months.

**Results:**

IGT and controls showed no statistically significant differences regarding preoperative variables like sex, gestational age, birth weight, syndromes, and EA type. However, IGT patients had a significantly longer esophageal GAP under boost pressure (4.5 vertebral bodies vs. 3.6, *p* = 0.019) at time of surgery. The analysis showed no statistical difference among the two groups about perioperative outcomes, ICU, or overall postoperative stay. No differences have been shown between IGT and controls during the follow-up regarding GERD, esophagitis, fundoplication, dysphagia, vocal cord paralysis, stenosis, and dilatations, auxologic data, need for anastomosis revision, oral aversion, and death.

**Conclusions:**

Isoperistaltic Gastric Tube is safe and effective even in LGEA patients with longer gaps, with good perioperative, post-operative and middle-term outcomes. This procedure may be considered as an alternative to avoid esophageal substitution when a primary anastomosis seems impossible for a residual gap after traction and growth techniques.

## Introduction

Despite clinical and technical progress, management of long-gap esophageal atresia (LGEA) remains challenging even for expert pediatric surgeons in dedicated centers (1). Controversy exists regarding the best surgical approach to repair LGEA, and several techniques have been developed to overcome difficulties (1–5). These include delayed primary anastomosis, lengthening procedures [e.g. serial pouch dilatation with bougienage, circular myotomy, esophageal flap, Foker procedure (traction suture esophageal lengthening) and Kimura technique (multistage extra thoracic esophageal elongation)], magnetic anastomosis and esophageal substitution with different techniques (gastric tube, gastric pull-up, jejunal, small bowel or colonic interposition) (1, 6, 7). Furthermore, well-designed comparative studies on short- and long-term outcomes are lacking (3). Among surgical options, gastric tube fashioning is not commonly used and reported. Theoretical advantages of this technique are the adequate and personalized graft length, the good blood supply, the ability to retain a tubular shape and the rapid food transit (3). Instead, possible speculative disadvantages may be long suture line, the high risk of leak and stricture, GERD and the risk of Barrett esophagus (3). However, limited evidence exists regarding perioperative and follow-up outcome after esophageal elongation with gastric tube in LGEA (1, 3). The last ERNICA Consensus Conference on the management of long gap esophageal atresia has placed the techniques of gastric tubulization among the issues to be priorities for future research (1).

Aim of the present study was to review our experience with isoperistaltic gastric tube, comparing perioperative and follow-up outcomes with other patients with LGEA approached with different surgical techniques to define the suitability and the role of this procedure.

## Methods

**Figure 1:**
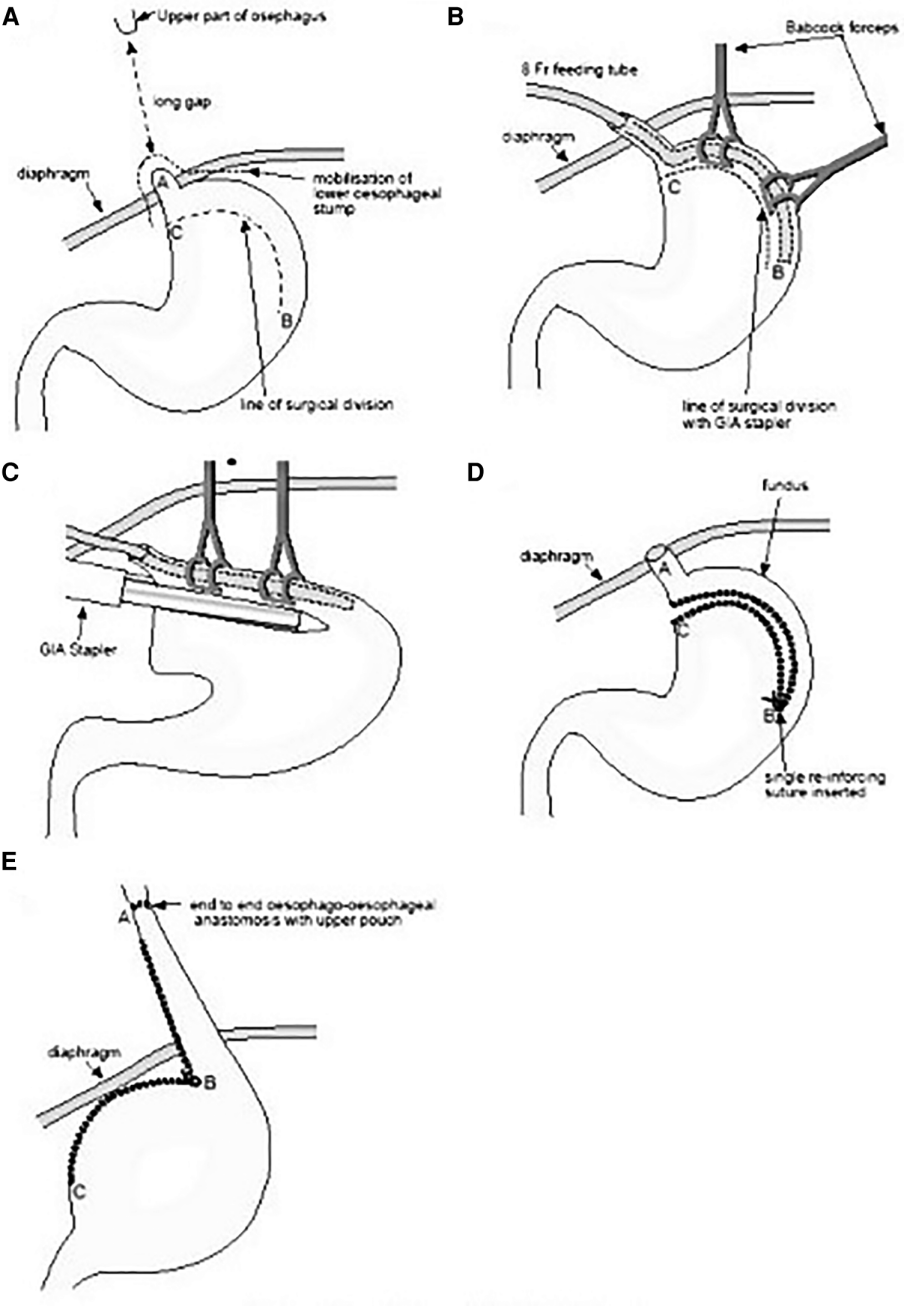
A–E: The technique of isoperistaltic gastric tubulization according to Collins (modified Scharli) is depicted and detailed step by step from A to E (image adapted and modified from: *Beasley SW, and Skinner AM*. (12)).

A retrospective analysis of all patients treated with IGT for LGEA in our tertiary center between 2010 and 2019 was performed. Patients were identified from a prospectively filled institutional database of esophageal atresia (EA). LGEA was defined according to the working group on LGEA of the International Network of Esophageal Atresia (Type A–B classification according to Gross and Ladd) (2). All patients selected for IGT were included in the study group. The IGT technique has been used as a “rescue procedure” with two indications: (1) after traction according to Foker or Kimura, when the residual gap was still partial but such as not to allow anastomosis (4 traction cases: 3 Foker, 1 Kimura); (2) in cases in which it was assessed at the time of initial surgery that the IGT would have allowed the anastomosis to be reached in a single stage, avoiding the long path of “traction and growth technique” (1 case). Consecutive cases treated for long gap esophageal atresia (with traction, 9 cases, and without traction, 1 case) in which anastomosis was performed were considered potential controls. Each case treated with IGT was then compared with two selected and matched controls, considering also the type of initial esophageal atresia (Type A–B classification according to Gross and Ladd). Clinical, radiological, and surgical data were revised, as well as follow-up and outcomes. The methodology for esophageal gap measurement under standardized boost pressure and general management of difficult esophageal atresia has been previously detailed (8). All patients have been followed-up for at least 24 months after surgery. The first 24 months of follow-up have been considered for the present study. Patients were excluded from the present study if lost at follow-up. The patients underwent a structured multidisciplinary follow-up according to ERNICA Consensus Conference and NASPGHAN/ESPGHAN guidelines (1, 9). Our follow-up program for esophageal atresia consists in prospective evaluation at defined time points which evaluate aspects related to several clinical disorders such as: growth, digestive, respiratory, skeletal and neurodevelopmental (10). Furthermore, to detect any specific growth delays, which can be exacerbated by perinatal surgical management and comorbidities, we carefully analyzed and compared the weight and height of the patients for post-surgical growth (z-score at 6 months, 12 months, and 24 months) consistently with our follow-up program. The isoperistaltic gastric tube has been created according to Collins (modified Scharli) technique (11, 12). The technique is summarized in Figure 1. Other surgical options used for control group included direct esophageal anastomosis under tension, Foker's traction and growth procedure, Kimura's traction and growth procedure, or combinations of the two. No patients in the IGT group had a cervical esophagostomy. Our main traction technique was Foker procedure. Extrathoracic Kimura technique was reserved to patients referred with a cervical esophagostomy. All the patients included in the present study underwent open minimal surgeries (8). Surgical complications have been reviewed, detailed and classified according to Clavien–Dindo (13). The indication to surgical therapy of GERD (fundoplication) in these patients were in accord with the recommendations of the North American Society for Pediatric Gastroenterology, Hepatology, and Nutrition (NASPGHAN) and the European Society for Pediatric Gastroenterology, Hepatology, and Nutrition (ESPGHAN) (9).

Categorical variables are reported as absolute and relative frequencies. For continuous variables a Kolmogorov–Smirnov test for normality was performed. Normal distribution continuous variables are reported as mean and standard deviation, non-normal distribution variables as median and range. Groups were compared using the *χ*^2^ test or the Fisher exact test for categorical variables, as appropriate. For non-normal distribution variables, differences between groups were established with a non-parametric test, *U* Mann–Whitney test. For normal distribution variables, differences between groups were established with a *T* Student test. All *p*-values were two-sided, and a value <0.05 was considered significant.

Present study received authorization for publication from the scientific board in the authors' institution. Due to the retrospective nature of the study, Institutional Review Board waived the need for informed consent.

## Results

During the 10-year study period, 204 patients with EA were managed at our institution. Among these, 43 (21%) were LGEA. Fifteen LGEA consecutively treated were enrolled into present study: 5 received IGT and 10 matched as controls. Demographic and perioperative outcomes considered were similar between IGT and controls groups, except for esophageal gap length, which was significantly longer in IGT patients (4.5 vs. 3.3 vertebral bodies, *p* = 0.019) (Table 1). Four patients were syndromic: three had VACTERL association, and one a deletion of 22q11.21. Three of these patients underwent a delayed esophageal anastomosis, while one needed a Foker procedure. Two syndromic patients (50%) and 8 non syndromic patients (72%) experienced major complications (Clavien–Dindo ≥IIIa). The complication rate between syndromic and non-syndromic patients did not show statistical difference (50% vs. 72%, *p* = 0.506).

**Table 1:** Demographic data of patients and preoperative variables.

	Control (*n* = 10)	IGT (*n* = 5)	*p*-value
Females	5 (50%)	2 (40%)	1.000
Mean gestational age (weeks) (SD)	34.7^a^ (±3.2)^b^	36.6^a^ (±2.4)^b^	0.265
Treated since birth	4 (40%)	2 (40%)	1.000
Mean birth weight (g) (SD)	2,127^a^ (±644)^b^	2,353^a^ (±478)^b^	0.501
Preterm (<37 weeks)	6 (60%)	3 (60%)	1.000
Syndromic	4 (40%)	0 (0%)	0.231
Gross			
A	6 (60%)	3 (60%)	1.000
B	4 (40%)	2 (40%)	1.000
Mean Gap (vertebral bodies) (SD)	3.6^a^ (±0.4)^b^	4.5^a^ (±0.8)^b^	**0** **.** **019**

Bold highlights denote the statistically significant value. ^a^Mean. ^b^Standard deviation.

Regarding the peri-operative and post-operative outcomes considered no difference was observed between IGT and controls. Indeed, the groups presented similar rate of elongation procedures (either Foker or Kimura operation), postoperative major complications (Clavien–Dindo ≥IIIa), leak, stenosis, ICU and overall post-operative hospital stay (Table 2).

**Table 2:** Surgical, operative, and post-operative variable.

	Control (*n* = 10)	IGT (*n* = 5)	*p*-value
Foker	4 (40%)	3 (60%)	0.608
Kimura	5 (50%)	1 (20%)	0.580
No traction & growth	1 (10%)	1 (20%)	1.000
Mean age at surgery (months) (SD)	9.6^a^ (±8.2)^b^	5.9^a^ (±2.8)^b^	0.356
≥1 major complication (Clavien–Dindo ≥IIIa)	6 (60%)	4 (80%)	0.600
Type of complications			
Leak	4 (40%)	1 (20%)	0.600
Stenosis	5 (50%)	1 (20%)	0.580
Mean postoperative ICU stay (days) (SD)	21.7^a^ (±18.9)^b^	17.6^a^ (±10.5)^b^	0.663
Mean postoperative stay (days) (SD)	84.5^a^ (±71.6)^b^	56.8^a^ (±22.1)^b^	0.420

^a^Mean. ^b^Standard deviation.

Finally, no significant differences were shown during the 24 months follow-up period (Table 3). All the auxological evaluations (weight and height of the patients at 6 months, 12 months, and 24 months) revealed similar growth after surgery for IGT and controls. Furthermore, the incidence of post-operative esophageal stenosis, requiring endoscopic dilation, were similar in the 2-groups at 6, 12 and 24 months. The study group and controls were similar for gastroesophageal reflux disease (GERD) (defined as percent time >7% and number of reflux >70/24 h at 24-h pH-Metry. At 2-year follow-up, no patient developed signs of metaplasia, dysplasia, or Barrett esophagus. Furthermore, similar rates of dysphagia and oral aversion, vocal cord paralysis, need for surgical re-intervention, and late mortality was found.

**Table 3:** Follow-up and outcomes.

	Control	IGT	*p*-value
Percent time >7% (24-h pH-Metry)	1/8 (12.5%)	2/5 (40%)	0.510
All reflux >70/24 h (24-h pH-Metry)	0/8 (0%)	0/5 (0%)	1.000
Fundoplication	5/10 (50%)	1/5 (20%)	0.580
Dysphagia	5/10 (50%)	3/5 (60%)	1.000
Esophagitis on histology	3/10 (30%)	2/5 (40%)	1.000
Vocal cord paralysis	1/10 (10%)	1/5 (20%)	1.000
Esophageal stenosis			
6-months	8/10 (80%)	3/5 (60%)	0.560
12-months	6/10 (60%)	1/5 (20%)	0.282
24-months	3/10 (30%)	2/5 (40%)	1.000
Esophageal endoscopic dilatations in 24 months (number)			
≥3	8/10 (80%)	3/5 (60%)	0.560
≥6	7/10 (70%)	2/5 (40%)	0.329
≥10	4/10 (40%)	2/5 (40%)	1.000
Endoscopic stent	1/10 (10%)	0/5 (0%)	1.000
Mean z-score weight (SD)			
6-months	−1.88^a^ (±1.8)^b^	−1.01^a^ (±1.2)^b^	0.370
12-months	−1.47^a^ (±1.4)^b^	−1.43^a^ (±0.6)^b^	0.955
24-months	−1.44^a^ (±1.1)^b^	−1.65^a^ (±0.4)^b^	0.718
Mean z-score height (SD)			
6-months	−2.47^a^ (±1.9)^b^	−1.26^a^ (±0.9)^b^	0.208
12-months	−0.90^a^ (±2.2)^b^	−1.86^a^ (±1.3)^b^	0.402
24-months	−0.79^a^ (±0.6)^b^	−1.11^a^ (±0.5)^b^	0.367
Surgical re-intervention	1/10 (10%)	1/5 (20%)	1.000
Oral aversion	2/10 (20%)	1/5 (20%)	1.000
Death	0 (0%)	0 (0%)	1.000

IGT, isoperistaltic gastric tube; FU, follow-up. ^a^Mean. ^b^Standard deviation.

## Discussion

Management of LGEA remains challenging even in dedicated centers. A recent ERNICA consensus statement defined delayed primary esophageal anastomosis as the ideal goal for LGEA repair when feasible, even under tension (1). Nonetheless, when direct primary anastomosis is unachievable, several different surgical options are available to fill the residual gap. Esophageal substitutions were proposed as standard techniques at the beginning, but later partially overcome by traction-growth and elongation techniques. On this regard, the gastric tubulizations can be considered an elongation of lower esophagus, a middle way between esophageal traction and esophageal replacement. The technique in fact, consists in a T-T esophago-esophageal anastomosis and not, as in true substitutions/replacement, in a colon/jejunum/gastro-esophageal anastomosis. For this reason, we cannot define IGT as a substitution technique, since all the available esophagus (proximal and distal) is used and not “replaced”. More strictly and more properly speaking, it could be made similar to a sliding hiatus hernia which is sometimes the result of traction even in patients in whom the gap could be bridged at primary surgery. Furthermore, the term “isoperistaltic”, simply indicates the “orientation” of the digestive tract used, without implying whether the segment used has an effective peristaltic wave. Consistently, the Gavriliu technique (unlike the IGP) is an “antiperistaltic” gastric tube (14). Similarly, colon replacement can be performed by orienting the colon as isoperistaltic or antiperistaltic (based on vascular supply needs) (1). Taking the concept of peristalsis to extremes to define the term “peristaltic” (iso or anti) one can even speculate that also the esophago-esophageal anastomosis would not be “peristaltic” being known the peristaltic anomaly of the distal esophageal tract which presents tertiary (or absent) peristalsis as reported in several studies. IGT allows the isoperistaltic elongation of the lower esophagus of adequate length and caliber, personalized on the gap of patient (6, 11, 12, 15–25). In our experience, we present five patients in whom this technique avoided an esophageal replacement. There were no significant differences between the perioperative and middle-term complications compared to controls. IGT cannot solve all problems of patients with LGEA. However, it may represent an option, in an extremely selected subgroup of LGEA with a residual gap after more “standard” procedures, to bridge it. It therefore seems possible to hypothesize that this technique can be considered in cases in which other traction and growth techniques have not allowed the gap to be completely bridged or in those in which IGT, due to a limited gap after the maximal dissection of the two esophageal stumps, can avoid the long and heavy surgical path of traction and growth. Furthermore, the use one IGT as primary instead of a rescue procedure could lead to speculate on its role to avoid the long path of traction and growth technique in selected case. Indeed, IGT cannot be considered “better” than the other most used ones, but a possible useful alternative that it does show similar outcomes and complications.

Consistently with our findings, IGT has been reported as a feasible and safe procedure even in selected cases where excessive tension is present (26). To date, there are few studies on IGT in pediatric population, especially LGEA. Moreover, there are crucial difficulties in comparing different series. To the authors' best knowledge only one series with a control group has been reported on IGT in LGEA (6). Lee et al. conducted a retrospective 25-year review comparing outcomes of delayed primary anastomosis vs. greater curvature isoperistaltic gastric tube for LGEA. Inconsistently with the INoEA definition of LGEA, those authors defined LGEA as the not-feasible immediate anastomosis with a consequent delay of esophageal repair. Similarly to our series, IGT patients showed longer gaps (mean 5.5 vertebrae, range 4–9) compared to delayed primary anastomosis (mean 3.9, range 2–6) (*p* = 0.004), but no difference in perioperative complications. However, Lee and co-workers found a higher rate of long-term complications (86%) for IGT patients, concluding that LGEA infants can be treated with IGT but the long-term follow-up is mandatory. Our experience on IGT shows no significant difference in morbidity when compared with other more used techniques. Nonetheless, we strongly agree with Lee and co-authors on the importance of prospective long-term follow-up evaluation in all EA patients, with special regards to LGEA ones. Similar recommendations were recently suggested in ERNICA consensus papers, focused on EA and LGEA infants (1). Patients' centralization, multidisciplinary approach, and involvement of patient organizations will provide the cornerstone for uniform treatment protocols and resultant optimized patient care.

Present work has some limitations, mainly related to the retrospective and monocentric nature of the study and the limited number of cases enrolled. The sample size must be interpreted in the light of the super selected group analyzed (cases with long gap esophageal atresia treated with this “rescue” technique in case of failure of more “standard” procedures).

Regardless possible limitations, present study adds insights into a technique not still widespread but possibly useful, in highly selected cases, to avoid esophageal replacement. Furthermore, the present experience suggests possible indications of isoperistaltic gastric tubulization and delineate criteria for selection of candidate patients. Further prospective multicenter studies are required.

## Conclusions

Isoperistaltic gastric tube was safe and effective as other techniques for highly selected cases of LGEA with a residual gap after more common procedures to bridge it. In LGEA when a delayed esophageal anastomosis is not achievable, with or without the use of traction and growth techniques, IGT creation can be considered in centers of expertise as a “rescue” technique to fill the gap, limiting esophageal replacement.

## Data Availability

The raw data supporting the conclusions of this article will be made available by the authors, without undue reservation.
